# Understanding Ancient Hominin Dispersals Using Artefactual Data: A Phylogeographic Analysis of Acheulean Handaxes

**DOI:** 10.1371/journal.pone.0007404

**Published:** 2009-10-14

**Authors:** Stephen J. Lycett

**Affiliations:** Department of Anthropology, University of Kent, Canterbury, United Kingdom; University College London, United Kingdom

## Abstract

**Background:**

Reconstructing the dispersal patterns of extinct hominins remains a challenging but essential goal. One means of supplementing fossil evidence is to utilize archaeological evidence in the form of stone tools. Based on broad dating patterns, it has long been thought that the appearance of Acheulean handaxe technologies outside of Africa was the result of hominin dispersals, yet independent tests of this hypothesis remain rare. Cultural transmission theory leads to a prediction of a strong African versus non-African phylogeographic pattern in handaxe datasets, if the African Acheulean hypothesis is to be supported.

**Methodology/Principal Findings:**

Here, this prediction is tested using an intercontinental dataset of Acheulean handaxes and a biological phylogenetic method (maximum parsimony). The analyses produce a tree consistent with the phylogeographic prediction. Moreover, a bootstrap analysis provides evidence that this pattern is robust, and the maximum parsimony tree is also shown to be statistically different from a tree constrained by stone raw materials.

**Conclusions/Significance:**

These results demonstrate that nested analyses of behavioural data, utilizing methods drawn from biology, have the potential to shed light on ancient hominin dispersals. This is an encouraging prospect for human palaeobiology since sample sizes for lithic artefacts are many orders of magnitude higher than those of fossil data. These analyses also suggest that the sustained occurrence of Acheulean handaxe technologies in regions such as Europe and the Indian subcontinent resulted from dispersals by African hominin populations.

## Introduction

Understanding the dispersal patterns of Plio-Pleistocene hominins is a major research focus in palaeoanthropology (e.g. [1]–[10]). Such reconstructions of hominin movements are essential for understanding the pattern of human evolution and for assessing evolutionary scenarios [5]–[8]. Given the fragmentary nature of the hominin fossil record, and the frequent controversies that surround the dates of key specimens, the reconstruction of hominin dispersal patterns is, however, often fraught with difficulty [1], [5]. One potential means of supplementing fossil evidence for dispersal events is to use archaeological evidence in the form of stone artefacts [3], [11]. Being inherently more resilient to decay than osseous material, fully exploiting the potential that these lithic remains might offer in order to address issues of palaeobiological relevance, is an important goal.

It is widely accepted that around 1.7–1.6 million years ago a new form of stone tool began to appear in sub-Saharan Africa, especially in eastern and southern regions [12]–[14]. These new stone artefacts – termed ‘handaxes’ – consisted of roughly triangular, teardrop, or ovate-shaped pieces of stone which were knapped bifacially (i.e. flakes were removed from opposite sides of the piece). By at least the Middle Pleistocene (i.e. ∼500 thousand years ago) such artefacts have a widespread distribution, occurring at sites in Europe, the Near East and the Indian subcontinent, and collectively this widely distributed technological phenomenon is referred to as the ‘Acheulean’ [15]. Since the oldest known examples of handaxe technology are known from eastern and southern Africa, it is a widely held assumption within palaeoanthropology that their appearance in more distant regions of the Old World is due to the dispersal of African populations who took knowledge of this technology with them [7], [11], [16], [17]. However, while such a scenario is broadly consistent with the available chronological data, formal and independent tests of this hypothesis remain rare.

In recent years it has been increasingly recognized that the manufacture of artefacts such as handaxes results from the process of social transmission of knowledge between individuals and across generations [18]–[21]. It is also been increasingly recognized that social transmission may be modeled as a mechanism of inheritance broadly analogous to that of genetic transmission [22]–[27]. This is not to say that these two inheritance mechanisms are identical in all respects. One obvious difference is that in the case of social transmission the ability to acquire information is not limited solely to copying biological parents; there is also the opportunity to copy more distantly related kin and unrelated individuals. Nevertheless, attention has increasingly been drawn to the fact that the evolution of cultural traditions involves a process of social inheritance, variation in the details of practice, and differential representation of given variants in subsequent generations (i.e. sorting due to various selection processes and cultural drift) (e.g.[28], [29]). One outcome resulting from recognition of this analogous process has been an increase in the application of population genetic and phylogenetic methods drawn from biology in order to understand the evolution of cultural phenomena, including artefacts (e.g.[10], [30]–[43]).

Lycett and von Cramon-Taubadel [10] recently exploited the analogy between social transmission and genetic transmission in order to test the African Acheulean dispersal hypothesis. Studies of both genetic and phenotypic data in humans have shown that when hominin taxa disperse over large distances there is a correlated reduction of within-group variance with increased distance from geographic source (e.g. [44]–[47]). This phenomenon has been termed the ‘serial founder effect’: quite literally serial bottlenecking due to the sequential reduction of within-group genetic variance as effective population sizes become progressively smaller with each dispersal event. Hence, using the serial founder effect model as a basis, Lycett and von Cramon-Taubadel [10] made the equivalent prediction that if the African Acheulean dispersal hypothesis is to be supported, then handaxe datasets should exhibit a decrease in within-assemblage variance with increased distance from sub-Saharan Africa. They tested this prediction using samples of Acheulean handaxes from sub-Saharan Africa, north Africa, the Near East, Europe and the Indian subcontinent. The analysis found statistically significant support for the serial founder effect model with ∼45–50% of within-assemblage variance explained by geographic distance from Africa. Hence, this analysis appeared to support the African Acheulean dispersal hypothesis.

A further basic prediction that might be derived from the African Acheulean dispersal hypothesis is that there should be a strong African versus non-African phylogeographic pattern to Acheulean datasets. The notion of using phylogeographic patterns to infer dispersal events can be traced back to Hennig's [48] concept of ‘Progression rule’. This concept proposed that the most plesiomorphic taxa in a cladogram would be situated in a hypothesized geographic centre of origin, while the more derived taxa would be those most distant from the putative center of origin. The simplistic nature of such a prediction has since been criticized, especially since vicariance events may disrupt patterns created by dispersals [49], [50]. However, the basic premise that dispersal events are capable of producing phylogeographic patterns remains sound. It is notable in this regard that phylogenetic studies of human genetic data reveal strong phylogeographic patterns, with African populations falling close to the root, as might be expected from the strong fit of such data to a serial founder effect model (e.g. [51]). As such, a conservative prediction of the African Acheulean dispersal hypothesis – especially in the light of Lycett and von Cramon-Taubadel's [10] results – is that we might expect a strong African versus non-African phylogeographic pattern in handaxe datasets.

Here, this phylogeographic prediction is tested using quantitative data taken on Acheulean handaxes and a phylogenetic method drawn from biology (maximum parsimony). Robustness of the phylogenetic pattern was assessed using a randomization procedure (phylogenetic bootstrapping), and the maximum parsimony tree was compared statistically with a model tree constrained by stone raw materials.

## Results

**Figure 1:**
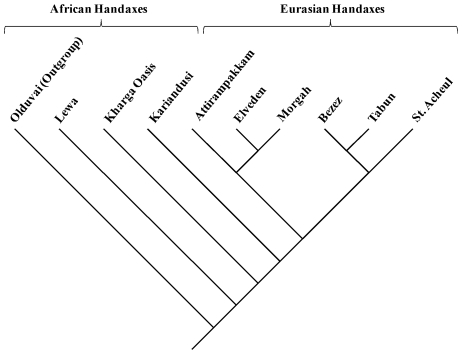
Maximum parsimony tree based on 66 characters (Tree length  =  1222).

Figure 1 shows the maximum parsimony tree returned by cladistic analysis of the handaxe dataset. As predicted by the African Acheulean dispersal hypothesis, this tree fits a phylogeographic pattern, with the African handaxe assemblages being situated close to the base of the tree (i.e. they are plesiomorphic), while the Eurasian handaxe assemblages occupy higher positions (i.e. they are derived relative to the African specimens).

**Figure 2:**
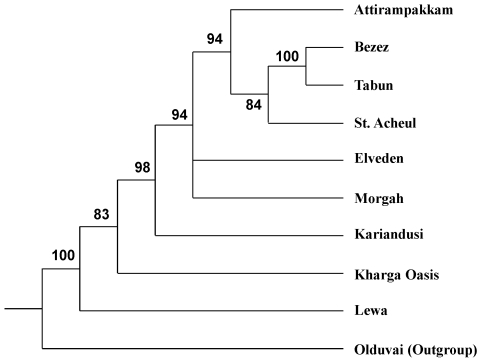
50% majority-rule consensus bootstrap tree (based on 10,000 bootstrap iterations).

The results of the bootstrap analysis strongly support the phylogeographic pattern indicated in the maximum parsimony tree (Figure 2). The node supporting a branching relationship between the African versus non-African handaxe assemblages is supported in 98% of the 10,000 bootstrap trees, while the node supporting the phylogenetic propinquity of all the non-African assemblages is supported in 94% of the bootstrap trees. Hence, this randomization analysis demonstrates that the predictions of the African Acheulean dispersal hypothesis are robustly supported by the data.

Table 1 reports the outcome of the Kishino-Hasegawa [52] test designed to assess the influence of stone raw materials on the maximum parsimony (MP) tree. Differences between the MP cladogram and the raw material model tree were found to be highly significant (p<0.0001). Hence, it does not appear that raw material has been a confounding factor in the phylogeographic test of the African Acheulean dispersal hypothesis.

**Table 1:** Results of K-H test (Tree 1  =  Maximum parsimony tree; Tree 2  =  Raw material model tree).

Tree	Length	Length difference	SD difference	*p*-value
1	1222	178	37.4	<0.0001
2	1400			

## Discussion

It has long been thought that the appearance of Acheulean handaxes outside of Africa is the result of hominin dispersals from that continent into Eurasia. Such thinking is based largely on the fact that the oldest examples of Acheulean handaxes appear in Africa, yet crucially, formal and independent tests of this hypothesis remain rare. Cultural transmission theory (e.g.[27]) and recent analyses of Acheulean data that utilize models drawn from population genetics [10] suggest a prediction for this African Acheulean dispersal hypothesis, which is testable using biological phylogenetic methods. That is, we may predict a strong African versus non-African phylogeographic pattern in handaxe datasets. Hence, if such a phylogeographic pattern were to be found and shown to be robust, this would provide an important line of support for the African Acheulean dispersal hypothesis.

Parsimony analyses of the Acheulean handaxe dataset, which includes samples from Africa, the Near East, Europe and the Indian subcontinent, produced a tree consistent with the phylogeographic prediction derived from the African dispersal hypothesis. Importantly, a randomization procedure (phylogenetic bootstrapping) provided further evidence that the major African versus non-African phylogeographic pattern depicted in the maximum parsimony (MP) tree is robust. Moreover, the MP tree was also shown to be statistically different from a comparative tree constrained by the raw materials used to manufacture the stone artefacts. This latter result demonstrates that raw material parameters (long known to be a potential influence on the form of stone tools) do not constitute a confounding factor in these analyses.

These results demonstrate that nested analyses of behavioural data, utilizing methods drawn from biology, have the potential to shed light on ancient hominin dispersals. This is an encouraging prospect for human palaeobiology since sample sizes for lithic artefacts are many orders of magnitude higher than those of fossil data. As noted earlier, an understanding of the dispersal patterns of ancient hominins is of major palaeobiological importance, and crucial toward a better understanding of human evolution [5], [6], [8]. Accordingly, the analyses reported here have important implications for palaeoanthropology.

Something of the frustration often encountered in determining hominin dispersal patterns is illustrated by Dennell's [53: 393] recent comment regarding the appearance of the Acheulean in India: “The absence of any hominin skeletal evidence from Early Pleistocene India makes it impossible to establish whether the Acheulean represents a colonization event”. Current evidence suggests that simple stone core and flake tools were first manufactured in eastern Africa around 2.6 million years ago [54], although the taxonomic identity of their manufacturers remains highly controversial [55]. Around 1 million years later, the first Acheulean handaxes make their appearance in eastern and southern Africa [13], [14]. Most commonly, the manufacture of these artefacts is attributed to *Homo ergaster* (or what some would call African *Homo erectus*), although there is something of around a 100–200 thousand year interval between the earliest appearance of this taxon in the African fossil record and the dates of the earliest Acheulean artefacts [56]. Prior to 1.4 million years ago, all reliable instances of stone tool occurrences outside of Africa are represented by cores and flake tools rather than handaxe technology [2], [17], [53].

The earliest reliable occurrence of Acheulean handaxes outside of Africa is that at ‘Ubeidiya in Israel during the Early Pleistocene (∼1.4 million years), although this has sometimes been considered a rather geographically proximate and temporary colonization episode [17], [57]. Recently, evidence for the presence of handaxes in southern Europe (Spain) as early as the terminal Early Pleistocene (∼900 thousand years ago) has been reported [58]. An Acheulean presence is also recorded during the early Middle Pleistocene (∼780 thousand years) at Gesher Benot Ya'aqov in Israel [11]. By ∼600 thousand years ago, further evidence for the Acheulean is known from southern Europe [59], [60], and it appears in northern Europe by at least 500 thousand years ago [61]. The situation concerning the Indian subcontinent is somewhat less secure, although on the basis of currently available dating evidence the Acheulean in this region is ≤ 800 thousand years old [53], [62].

The analyses reported here support the hypothesis that the widespread appearance of Acheulean handaxes in areas such as India and Europe was the result of an external colonization process by African hominin populations. Given such a suggestion, it is interesting to note that in the bootstrap analysis reported here, only the relationships between handaxes from Elveden (United Kingdom) and Morgah (Pakistan) and the other Eurasian handaxe assemblages were unresolved in the bootstrap consensus tree (i.e. supported at a level of less than 50%). This latter result indicates that it is relationships between the northern European and South Asian assemblages that are most unclear, while the major pattern of branching between African versus non-African assemblages is robustly supported. This would also support the idea that the widespread and sustained occurrence of the Acheulean in Europe and the Indian subcontinent was the result of the same dispersal process from Africa [8], which on the basis of current evidence, intensified during the terminal Early Pleistocene and early Middle Pleistocene.

## Materials and Methods

Data for a total of 72 quantitative characters were collected for a series of handaxes from ten localities distributed across Africa, Europe, the Near East and the Indian subcontinent (total *n* = 255 handaxes) (Table 2). The 72 characters recorded for each handaxe are listed in Table S1. Detailed descriptions of these quantitative characters have previously been provided elsewhere (e.g. [39], [63], [64]). In order to ensure that morphometric data emphasize shape information rather than mere size differences between artefacts (the latter of which would tend to reflect initial raw material size rather than socially transmitted information regarding shape and/or socially transmitted factors affecting final shape) Characters 1–48 (Table S1) were size adjusted via the geometric mean [65]. In contrast to alternative methods of size adjustment, it has been shown that the geometric mean method effectively equalizes the volumes of specimens while preserving relevant shape information [66]. For the purposes of phylogenetic analysis, the quantitative characters were coded into discrete character states via the statistical procedure of divergence coding [67] (see Supplementary Text S1).

**Table 2:** Operational Taxonomic units employed in analyses.

Locality	*n*	Raw material
Attirampakkam, India	30	Quartzite
Bezez Cave (Level C), Adlun, Lebanon	30	Chert
Elveden, Suffolk, UK	24	Chert
Kariandusi, Kenya	30	Lava
Kharga Oasis (KO10c), Egypt	17	Chert
Lewa, Kenya	30	Lava
Olduvai Gorge (Bed II), Tanzania	13	Quartz, lava
Morgah, Pakistan	21	Quartzite
St Acheul, France	30	Chert
Tabun Cave (Ed), Israel	30	Chert

Total *n* = 255 handaxes.

Cladistic (parsimony) analyses were used to determine the strength of phylogeographic patterning in the Acheulean data. Cladistics is one of the main phylogenetic methods used in biology (e.g. [68]–[74]). As noted earlier, in recent years it has also been increasingly used in anthropology and archaeology, where gaining an understanding of the phylogenetic relationships between socially transmitted traditions is also important (e.g. [30]–[34], [36]–[40], [75]). Cladistic analysis has some advantages when using archaeological material since it does not rely on dating information to reconstruct phylogenetic relationships [33], [76]. Hence, even when chronological information is incomplete, phylogenetic patterns may still be determined.

A fundamental requirement of cladistic analysis is that characters are independent of one another in order to reduce redundant information [77], [78]. Following Nadel-Roberts and Collard [79], characters 1–64 (Table S1) were screened for statistically significant levels of correlation via Pearson product-moment analyses. Thereafter, where data for any two characters were found to be significantly (p≤0.05) correlated in all taxonomic units, one of these characters was removed from the character matrix. When any two variables are correlated in this manner, it is arbitrary which of these is removed in order to reduce this redundancy of information. Here, a randomization procedure was employed to select the character. Following this procedure, six characters were removed from the character matrix (Characters 7, 11, 16, 35, 40, 43: Table S1) leaving a total of 66 characters for the analyses.

The cladistic analyses were performed using PAUP* 4.0 [80]. The branch-and-bound search algorithm was used to undertake the analysis, which is guaranteed to find the most parsimonious cladogram(s) for a given character matrix [76]. Since all characters are quantitative, and may therefore be expected to have evolved serially [81], all characters were treated as ordered and freely reversing [82]. All characters were given equal weight in the analysis. In order to determine the direction (or ‘polarity’) of character state changes, the method of outgroup comparison was utilized [76]. The handaxes from Olduvai Gorge (Bed II), Tanzania were used for this purpose, since being in the region of 1.4–1.2 million years old, they are among some of the oldest known examples of Acheulean technology [12], [83] and are therefore most likely to be informative regarding plesiomorphic character states [84:58–59].

Following identification of the most parsimonious cladogram(s) a bootstrap analysis was undertaken in order to assess the robustness of the relationships indicated [76], [85]. The bootstrap procedure involves randomly resampling characters with replacement in order to create a large number of pseudo-replica data matrices with the same number of characters and character states as the original. Thereafter, each pseudo-replica character matrix is subjected to parsimony analysis. Bootstrap results are typically presented in the form a majority-rule consensus tree, with the percentage of bootstrapped trees supporting particular branching relationships indicated at the appropriate branching points of the majority-rule tree. Following recent biological applications of phylogenetic bootstrapping (e.g. [86], [87]) 10,000 pseudo-replica character matrices were subjected to parsimony analysis.

An additional post-hoc analysis was undertaken to assess the influence of the stone raw materials (used to manufacture the handaxes) on the analyses. Table 2 shows that the handaxes analysed here were made on a range of different raw materials. For some time, archaeologists have recognized that differences in the physical properties of different stone raw materials (e.g. hardness, brittleness, granularity) may have an influence on the final form of stone tools [88]. Hence, raw material factors might potentially be a confounding factor when attempting to infer phylogenetic patterns in stone artefacts. Here, the influence of raw material on the results was assessed statistically using the Kishino and Hasegawa (K-H) [52] test. This test uses the standard deviation of changes in each character and the *t*-statistic to determine if the maximum parsimony cladogram is significantly different from that of a comparative tree, the structure of which is known to be constrained by raw material factors. If the trees are statistically different, the null hypothesis of ‘no difference’ may be rejected (α = 0.05) (e.g. [89], [90]). In essence, the K-H test allows the maximum parsimony cladogram to be compared to a ‘model’ tree and facilitates the possibility of statistically rejecting the model (e.g. that the maximum parsimony tree is entirely the result of raw material properties).

**Figure 3:**
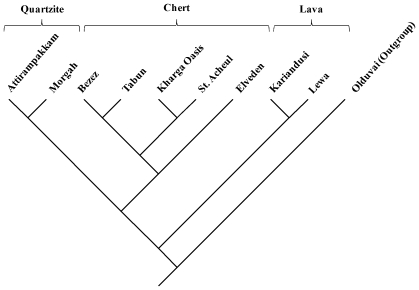
Raw material model tree.

In order to implement the K-H test, a model tree was built by first constructing a constraint tree reflecting pure raw material groups (i.e. taxonomic units of identical raw material were linked together in a multifircating clade). This constraint tree was constructed manually in MacClade 4.02 [91]. Subsequently the constraint tree was imported into PAUP*4.0 and a parsimony analysis conducted to find the cladogram most consistent with these raw material constraints. This cladogram (Figure 3) subsequently became the model tree used for the purposes of the statistical test.

## Acknowledgements

For important comments and discussions I am grateful to Parth Chauhan, Mark Collard, John Gowlett, Chris Norton, Mike O'Brien, Stephen Shennan, Noreen von Cramon-Taubadel, and the anonymous reviewers.

## Supporting Information

Table S1 Character list
(0.06 MB DOC)

Text S1 Methods
(0.04 MB DOC)
