# Supplementary material for: Understanding Ancient Hominin Dispersals Using Artefactual Data: A Phylogeographic Analysis of Acheulean Handaxes
Source: PLoS One. 2009 Oct 14;4(10):e7404. doi: 10.1371/journal.pone.0007404 (PMC2756619; doi:10.1371/journal.pone.0007404)
Supplement: Table S1 — Character list (0.06 MB DOC) [file pone.0007404.s001.doc]

**Table S1:** Characters employed in cladistic analyses. Six characters (i.e. characters 7, 11, 16, 35, 40, and 43) were not employed due to integration (see Lycett, 2009 for further details) leaving a total of 66 characters for the analyses.

| 1. Core left width at 10% of length |
| --- |
| 2. Core left width at 20% of length |
| 3. Core left width at 25% of length |
| 4. Core left width at 30% of length |
| 5. Core left width at 35% of length |
| 6. Core left width at 40% of length |
| 7. Core left width at 50% of length |
| 8. Core left width at 60% of length |
| 9. Core left width at 65% of length |
| 10. Core left width at 70% of length |
| 11. Core left width at 75% of length |
| 12. Core left width at 80% of length |
| 13. Core left width at 90% of length |
| 14. Core right width at 10% of length |
| 15. Core right width at 20% of length |
| 16. Core right width at 25% of length |
| 17. Core right width at 30% of length |
| 18. Core right width at 35% of length |
| 19. Core right width at 40% of length |
| 20. Core right width at 50% of length |
| 21. Core right width at 60% of length |
| 22. Core right width at 65% of length |
| 23. Core right width at 70% of length |
| 24. Core right width at 75% of length |
| 25. Core right width at 80% of length |
| 26. Core right width at 90% of length |
| 27. Core length distal at 10% of width |
| 28. Core length distal at 20% of width |
| 29. Core length distal at 25% of width |
| 30. Core length distal at 30% of width |
| 31. Core length distal at 40% of width |
| 32. Core length distal at 50% of width |
| 33. Core length distal at 60% of width |
| 34. Core length distal at 70% of width |
| 35. Core length distal at 75% of width |
| 36. Core length distal at 80% of width |
| 37. Core length distal at 90% of width |
| 38. Core length proximal at 10% of width |
| 39. Core length proximal at 20% of Width |
| 40. Core length proximal at 25% of Width |
| 41. Core length proximal at 30% of Width |
| 42. Core length proximal at 40% of Width |
| 43. Core length proximal at 50% of Width |
| 44. Core length proximal at 60% of Width |
| 45. Core length proximal at 70% of Width |
| 46. Core length proximal at 75% of Width |
| 47. Core length proximal at 80% of Width |
| 48. Core length proximal at 90% of Width |
| 49. Coefficient of surface curvature 0–180° |
| 50. Coefficient of surface curvature 90–270° |
| 51. Coefficient of surface curvature 45–225° |
| 52. Coefficient of surface curvature 135–315° |
| 53. Coefficient of edge-point undulation |
| 54. Index of symmetry |
| 55. Maximum width/width at orientation |
| 56. Maximum length/length at orientation |
| 57. Nuclei outline length |
| 58. Area of largest flake scar |
| 59. CV of complete flake scar lengths |
| 60. CV complete flake scar widths |
| 61. Total number of complete (i.e. untruncated) flake scars |
| 62. Total number of negative flake scars |
| 63. Number of flakes removed superior and in contact with outline of nucleus |
| 64. Number of non-feather terminations |
| 65. % Cortex 1st superior quadrant |
| 66. % Cortex 2nd superior quadrant |
| 67. % Cortex 3rd superior quadrant |
| 68. % Cortex 4th superior quadrant |
| 69. % Cortex 1st inferior quadrant |
| 70. % Cortex 2nd inferior quadrant |
| 71. % Cortex 3rd inferior quadrant |
| 72. % Cortex 4th inferior quadrant |
