# Supplementary material for: Understanding Ancient Hominin Dispersals Using Artefactual Data: A Phylogeographic Analysis of Acheulean Handaxes
Source: PLoS One. 2009 Oct 14;4(10):e7404. doi: 10.1371/journal.pone.0007404 (PMC2756619; doi:10.1371/journal.pone.0007404)
Supplement: Text S1 — Methods (0.04 MB DOC) [file pone.0007404.s002.doc]

**Supplementary Text S1**

**Quantitative handaxe characters used in the analyses**

Data for a total of 72 characters were collected for all 255 handaxes employed in the analyses (Table S1). Morphometric data were collected via use of a Crossbeam Co-ordinate Caliper (Lycett et al., 2006). As well as capturing specific aspects of artefact shape (in plan and section) and the position of cortex, several characters (i.e. 58–64) relate to additional ‘technological’ attributes such as consistency (CV) in the length and width of complete flake scars, the number of non-feather terminations, total number of negative flake scars, and the total number of untruncated (i.e. complete) flake scars. Details of the characters employed and the protocols used to acquire them have previously been described in detail elsewhere (Lycett et al., 2006; Lycett, 2007a, 2007b). Variables 1–48 listed in Table S1 (i.e. Euclidean distance variables) were size-adjusted by the geometric mean method (Jungers et al., 1995; Lycett et al., 2006) in order to remove the confounding effects of isometric differences in scale between various finished artefacts and initial blank form sizes. The geometric mean method of size-adjustment equalizes the volumes of the specimens while maintaining overall shape information (Falsetti et al., 1993; Jungers et al., 1995).

**Divergence coding of characters for maximum parsimony analysis**

It is sometimes suggested that quantitative (i.e. continuous) characters are fundamentally different from ‘discrete’ qualitative characters and should be excluded from phylogenetic analyses (e.g., Crisp and Weston, 1987; Pimentel and Riggins, 1987; Cranston and Humphries, 1988). However, such a position has been rebutted by numerous authors (e.g., Baum, 1988; Chappill, 1989; Thiele, 1993; Rae, 1998; Swiderski et al., 1998; MacLeod, 2002) who have shown that qualitative characters are invariably points along a continuous scale of variation yet, confusingly, are described in a manner that merely implies ‘discreteness’ (e.g., ‘moderately curved’, ‘curved’, ‘highly curved’, etc.). As MacLeod (2002: 103) has wryly noted, even a seemingly unambiguous and so-called ‘discrete’ character such as colour, which is sometimes used by those who criticise the use of metric characters (e.g., Pimentel and Riggins, 1987), is actually a ratio-scale variable based on the frequency spectrum of reflected light. Hence, there is no justification for rejecting the use of metric characters on the basis of an assumed fundamental difference between the nature of qualitative and quantitative statements about morphology. In addition, quantitative characters offer several advantages over qualitative characters in terms of facilitating size-adjustment so that the confounding effects of size may be diminished (Rae, 2002), and the potential to screen characters for integration via statistical procedures (Nadel-Roberts and Collard, 2005). However, it is in regard to the issue of increasing the repeatability of character coding and reducing ambiguity that quantitative characters offer particular advantages. For instance, while a character such as ‘length’ could arbitrarily be divided into ‘long’, ‘medium’ and ‘short’ character states via qualitative assessment, variations of such a character will be evident both within and between OTUs. If such states overlap, or even come close to overlapping, character state assignation will inevitably become increasingly subjective (Rae, 1998). Conversely, the use of morphometric data allows character state assignations to be made on a non-arbitrary basis via statistical analysis of character variation and difference, even in the event of some degree of overlap (e.g., Thorpe, 1984). It is for these reasons that continuous characters have become increasingly used in wide range of biological phylogenetic analyses (e.g., Simonovic, 1999; Collard and Wood, 2000; Davis et al., 2001; Strait and Grine, 2004; Hibbitts and Fitzgerald, 2005; Lycett and Collard, 2005), as well archaeological phylogenetics (O’Brien et al., 2001; Buchanan and Collard, 2007; Lycett, 2007b).

Several procedures have been proposed for converting morphometric data into discrete character states (e.g., Mickevich and Johnson, 1976; Simon, 1983; Thorpe, 1984; Archie, 1985; Baum, 1988; Thiele, 1993). However, as noted by Rae (1998), the most robust coding methods are those that assign character codes in a non-arbitrary manner on the basis of statistical inference. Indeed, as Richter (2005) has recently argued, the analysis of homology is a two-stage process, whereby putative morphological homology (i.e. correspondence of form) must be assayed prior to a test of phylogenetic homology using parsimony. Hence, a procedure termed divergence coding (Thorpe, 1984) was chosen as the means of character coding here because, in contrast to many alternative coding methods, it assigns character states on the basis of statistical analyses rather than arbitrary decisions, or untested assumptions of homology in the case of overlapping data. Thorpe (1984: 252) notes that divergence coding has the advantage of being able to reflect relatively small differences between OTUs, and the number of character states is related directly to the extent of evolution within the character. Divergence coding has been employed in a variety of phylogenetic contexts (e.g., fish [Simonovic, 1999], dung beetles [Davis et al., 2001] and snakes [Hibbitts and Fitzgerald, 2005]), but particularly in the cladistic analysis of primate morphological data (e.g., Collard and Wood, 2000; Collard and Wood, 2001; Young, 2003; Lycett and Collard, 2005; Nadel-Roberts and Collard, 2005). Divergence coding is very similar to Simon’s (1983) method of homogenous subset coding, which, on occasion (e.g., Strait and Grine, 2004), has been used as a direct substitute for divergence coding due to the virtually identical manner in which the two methods assign character states to taxonomic units.

The divergence coding method proceeds as follows. The mean values of each character are placed in ascending order, and a taxon-by-taxon matrix compiled for each of the characters to be coded. Cells in the top row of each matrix are arranged such that taxon means decrease from left to right, while in the first column they decrease from the top to the bottom. Thereafter, each cell of the matrix is assigned a score of −1, +1 or 0, depending upon the outcome of the statistical comparisons. Where the mean of the taxon in the column is significantly lower than that of the taxon in the corresponding row, a score of −1 is assigned. Where the mean of the taxon in the column is significantly higher than that of the corresponding row, a score of +1 is assigned. When differences between taxon means are not statistically significant, a score of 0 is assigned. Once this procedure is completed, the total score for each column (i.e. the sum of every 0, −1, and +1) is computed. Lastly, the relevant integer is added to each taxon total to ensure that each score is positive. The first ten character states (from lowest to highest) may be coded as 0–9. Character states from 11–*n* may be coded as letters (e.g., A–Z).

In coding the data set, a one-way analysis of variance (ANOVA) (α ≤0.05) with post-hoc least significant difference (LSD) pairwise comparisons was employed to test for statistical significance. In the case of the post-hoc LSD tests, there is no requirement to lower the critical alpha level (p-value) below 0.05, if the initial ANOVA is significant (Dytham, 2003, p. 116) (if the initial ANOVA is not significant, the character can be regarded as uninformative and may be discarded). Bonferroni correction procedures were not applied in the pairwise comparisons since such a procedure leads to elevated type II errors (Perneger, 1998; Nakagawa, 2004). In a phylogenetic analysis, elevated type II errors are especially problematic since this will lead to more false similarities (i.e. homoplasies) being incorporated into the data set (Lycett and Collard, 2005: 625), and divergence coding is regarded as a conservative approach to character coding for this very reason (Young, 2003: 446). Since ANOVA assumes a normal distribution (Sokal and Rohlf, 1995), a Kolmogorov–Smirnov (K–S) test was used to establish that data were normally distributed (p ≤0.05). Where data for a given character were found to be significantly divergent from normal, the data were logarithmically transformed (log*_e_*) and re-tested. During the ANOVA, Levene’s test for homogeneity of variances (p ≤0.05) was employed to ensure that data met this assumption. Any characters that were found not to be normally distributed following logarithmic transformation were subjected to a non-parametric Kruskal–Wallis (KW) test (p ≤0.05) with pairwise Mann–Whitney U-test comparisons. Again, following Dytham (2003: 121), the logic behind this procedure is that if the initial Kruskal–Wallis test is significant, there is no requirement to lower the critical p-value below 0.05 for the pairwise comparisons. This procedure was used to code the cortex characters (i.e. Characters 65–72). The ANOVA, LSD, Levene’s test, Kruskal–Wallis test, pairwise Mann–Whitney U-test and K–S tests were undertaken in SPSS v.12.0.1, with subsequent aspects of the coding procedure undertaken using the spreadsheet facilities of Microsoft Excel. Character matrices were subsequently written as text files in NEXUS format for analysis (Swofford, 1998).

**REFERENCES not included with article text**

Archie, J. 1985 Methods for coding variable morphological features for numerical taxonomic analysis. *Systematic Zoology* **34**, 326-345.

Baum, B. 1988 A simple procedure for establishing discrete characters from measurement data, applicable to cladistics. *Taxon* **37**, 63-70.

Chappill, J. 1989 Quantitative characters in phylogenetic analysis. *Cladistics* **5**, 217-234.

Collard, M. & Wood, B. 2000 How reliable are human phylogenetic hypotheses? *Proceedings of the National Academy of Sciences USA* **97**, 5003-5006.

Collard, M. & Wood, B. 2001 Homoplasy and the early hominid masticatory system: inferences from analyses of extant hominoids and papionins. *Journal of Human Evolution* **41**, 167-194.

Cranston, P. & Humphries, C. 1988 Cladistics and computers: a chironomid conundrum? *Cladistics* **4**, 72-92.

Crisp, M. & Weston, P. 1987 Cladistics and legume systematics, with an analysis of the Bossiaeeae, Brongniartieae and Mirbelieae. In *Advances in Legume Systematics, Part 3* (ed. C. Stirton), pp. 65-130. Kew: Royal Botanical Gardens.

Davis, A. L. V., Scholtz, C. H. & Harrison, J. D. G. 2001 Cladistic, phenetic and biogeographical analysis of the flightless dung beetle genus, *Gyronotus* van Lansberge (Scarabaeidae: Scarabaeinae), in threatened eastern Afrotropical forests. *Journal of Natural History* **35**, 1607-1625.

Dytham, C. 2003 *Choosing and Using Statistics: A Biologist's Guide*. Oxford: Blackwell Science.

Hibbitts, T. J. & Fitzgerald, L. A. 2005 Morphological and ecological convergence in two nactricine snakes. *Biological Journal of the Linnean Society* **85**, 363-371.

Lycett, S. J. & Collard, M. 2005 Do homoiologies impede phylogenetic analyses of the fossil hominids? An assessment based on extant papionin craniodental morphology. *Journal of Human Evolution* **49**, 618-642.

MacLeod, N. 2002 Phylogenetic signals in morphometric data. In *Morphology, Shape and Phylogeny* (ed. N. MacLeod & P. L. Forey), pp. 100-138. London: Taylor & Francis.

Mickevich, M. & Johnson, M. 1976 Congruence between morphological and allozyme data in evolutionary inference and character evolution. *Systematic Zoology* **25**, 260-270.

Nakagawa, S. 2004 A farewell to Bonferroni: the problems of low statistical power and publication bias. *Behavioral Ecology* **15**, 1044-1045.

Perneger, T. V. 1998 What's wrong with Bonferroni adjustments. *British Medical Journal* **316**, 1236-1238.

Pimentel, R. & Riggins, R. 1987 The nature of cladistic data. *Cladistics* **3**, 201-209.

Rae, T. C. 1998 The logical basis for the use of continuous characters in phylogenetic systematics. *Cladistics* **14**, 221-228.

Richter, S. 2005 Homologies in phylogenetic analyses − concept and tests. *Theory in Biosciences* **124**, 105-120.

Simon, C. 1983 A new coding procedure for morphometric data with an example from periodical cicada wing viens. In *Numerical Taxonomy* (ed. J. Felsenstein), pp. 378-383. Berlin: Springer-Verlag.

Simonovic, P. D. 1999 Phylogenetic relationships of Ponto-Caspian gobies and their relationship to the Atlantic-Mediterranean Gobiinae. *Journal of Fish Biology* **54**, 533-555.

Sokal, R. R. & Rohlf, F. J. 1995 *Biometry*. New York: W.H. Freeman & Co.

Strait, D. S. & Grine, F. E. 2004 Inferring hominoid and early hominid phylogeny using craniodental characters: the role of fossil taxa. *Journal of Human Evolution* **47**, 399-452.

Swiderski, D. L., Zelditch, M. L. & Fink, W. L. 1998 Why morphometrics is not special: coding quantitative data for phylogenetic analysis. *Systematic Biology* **47**, 508-519.

Thiele, K. 1993 The holy grail of the perfect character: the cladistic treatment of morphometric data. *Cladistics* **9**, 275-304.

Young, N. M. 2003 A reassessment of living hominoid postcranial variability: implications for ape evolution. *Journal of Human Evolution* **45**, 441-464.
